# Biomarkers of blood–brain barrier and neurovascular unit integrity in human cognitive impairment and dementia

**DOI:** 10.1002/alz.70104

**Published:** 2025-03-27

**Authors:** Scott R. French, Briana P. Meyer, Juan C. Arias, Swati Rane Levendovzsky, Craig C. Weinkauf

**Affiliations:** ^1^ Division of Vascular Surgery University of Arizona Tucson Arizona USA; ^2^ Department of Radiology, Integrated Brain Imaging Center University of Washington Medical Center Seattle Washington USA

**Keywords:** biomarker, blood‐based biomarkers, blood–brain barrier, cognitive impairment, cerebrospinal fluid biomarkers, imaging biomarkers, neurodegeneration, neurovascular unit

## Abstract

Blood–brain barrier (BBB) dysfunction is recognized as an early step in the development of Alzheimer's disease and related dementias (ADRD). Biomarkers are needed to monitor BBB integrity over time, better understand the role of the BBB in neurodegeneration, potentially help define long‐term ADRD risk, and monitor effects of therapeutics. In this review, we discuss the current biomarkers used to detect human BBB dysfunction in the context of cognitive decline and dementia. We also discuss promising candidate fluid biomarkers to detect BBB dysfunction in blood.

**Highlights:**

- BBB permeability occurs during normal aging and is further exacerbated in ADRD.
- In this review, we discuss in vivo imaging and CSF biomarkers of BBB dysfunction currently used in the setting of aging and ADRD in humans.
- We also review promising candidate blood‐based biomarkers that may represent BBB dysfunction.

## 1 THE BLOOD‐BRAIN BARRIER AND ITS RELEVANCE IN ADRD PATHOPHYSIOLOGY

**FIGURE 1 alz70104-fig-0001:**
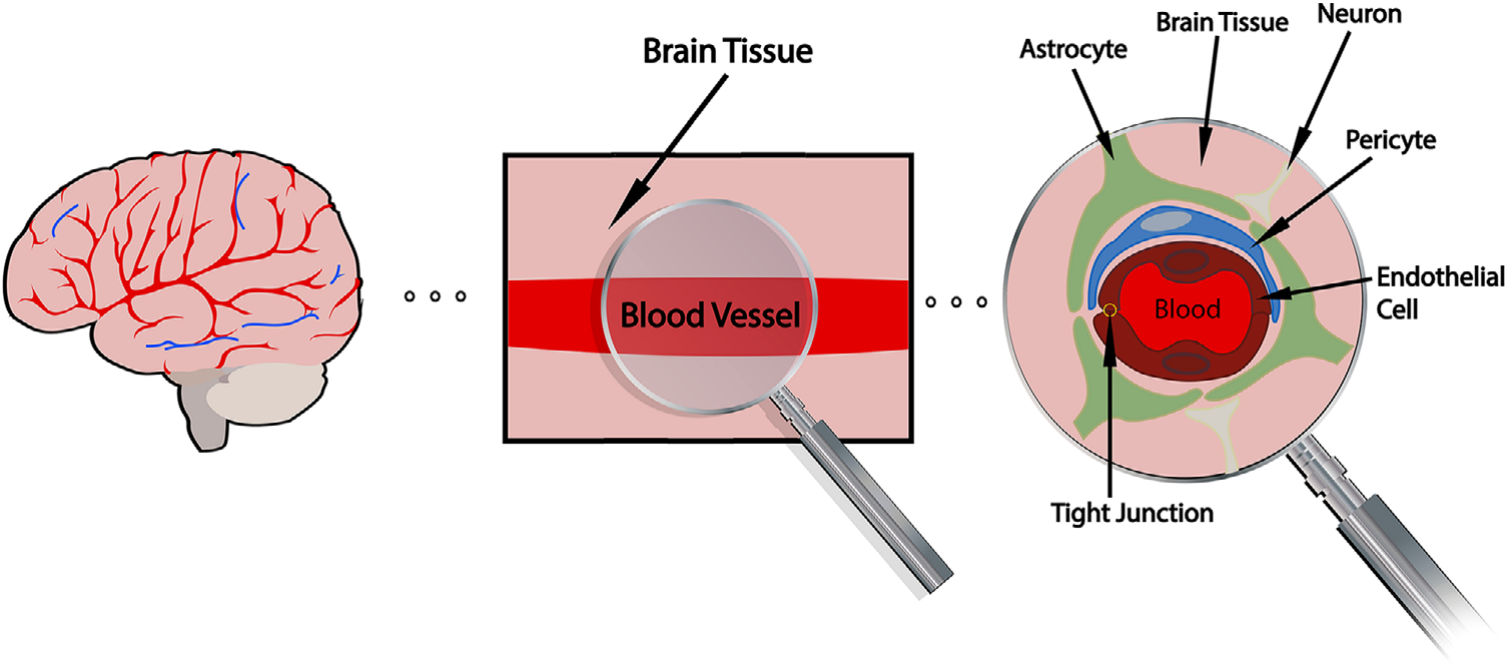
Schematic representation of blood–brain barrier (BBB). The BBB is a selective barrier in capillaries of the brain,^2^ also known as the neurovascular unit (NVU). It is composed of pericytes (blue), astrocytes (green), endothelial cells (dark red) connected by tight junctions (circled in yellow), and neurons (tan). The key differences between capillaries in the brain and the rest of the body are that brain capillary endothelial cells are continuously joined by tight junctions and lack fenestrations.^1^ Brain capillaries are surrounded by astrocyte endfeet, providing a direct link between blood vessels and neurons of the central nervous system (CNS). Pericytes are roughly 100× more abundant in capillaries of the CNS compared to the rest of the body.^3^, ^4^

The blood–brain barrier (BBB) is a semipermeable and highly selective barrier that regulates the flow of ions, molecules, and cells between the bloodstream and cerebral parenchyma.^1^, ^2^ The integrity of the BBB is attributed to the complex interactions of its neuronal and vascular components, known collectively as the neurovascular unit (NVU).^3^, ^4^ The NVU consists of continuous endothelial cells connected by tight junctions and surrounded by pericytes and astrocytic endfeet, and includes neurons and microglia (Figure 1).

**FIGURE 2 alz70104-fig-0002:**
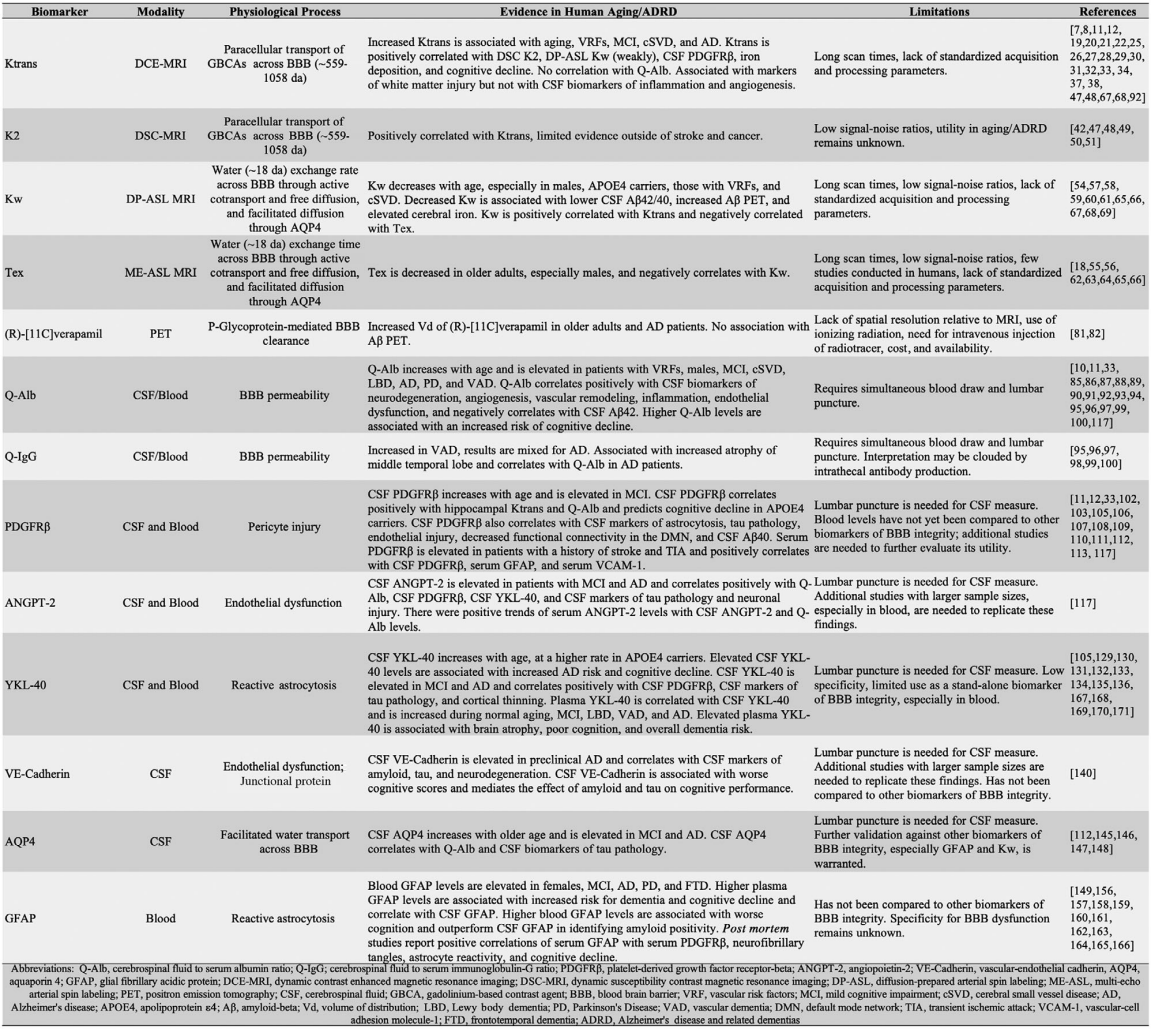
Summary of in vivo biomarkers of blood–brain barrier integrity in humans.

An intact BBB allows for O_2_ and other small hydrophobic molecules to freely diffuse across the membrane, while water, glucose, and ion flux is largely regulated by specific transporters or channels.^5^ BBB dysfunction is characterized by a disruption of one or more cell types within the NVU, resulting in impaired clearance of neurotoxic compounds (such as amyloid beta [Aβ] peptides and iron), impaired transporter function, and increased cerebral infiltration of peripheral immune cells, which can promote neuroinflammation.^6^ While some increased permeability of the BBB occurs in normal aging,^7^ it can be exacerbated in vascular conditions like hypertension or diabetes.^8^, ^9^, ^10^ This dysfunction allows potentially harmful substances to cross into the brain, triggering inflammatory responses and oxidative stress that can damage neurons and glial cells and subsequently promote the development of Alzheimer's disease and related dementias (ADRD).^11^, ^12^, ^13^ BBB dysfunction is an early step of ADRD progression, occurring prior to morphological changes in the hippocampus and Aβ/neurofibrillary tangle (NFT) accumulation and is worsened by the *APOE* ε4 allele independently of pathological markers of AD or vascular risk factors.^11^, ^12^ Early detection of BBB dysfunction could lead to innovative strategies to avert ADRD onset and progression in high‐risk individuals. For these reasons, biomarkers to measure BBB integrity are critically needed in both clinical and research settings.^14^ In this review, we discuss the current state of in vivo imaging and fluid biomarkers of BBB dysfunction in human subjects and highlight their utility and limitations and the extent to which each biomarker may be reflective of different subtypes of BBB dysfunction. A summary of the biomarkers discussed is shown in Figure 2.

## 2 IMAGING BIOMARKERS

### 2.1 Magnetic resonance imaging (MRI)

BBB integrity is measured by MRI in humans through quantification of BBB regional permeability using both contrast‐ and non‐contrast‐based MRI techniques. Contrast‐based methods use intravenous gadolinium‐based contrast agents (GBCAs) with molecular sizes too large to cross an intact BBB (∼559 to 1058 da).^15^ GBCAs can cross the BBB through paracellular transport when damage occurs to junctional complexes (tight, adherens, gap junctions) on the endothelium.^16^ Non‐contrast‐based methods measure permeability by magnetically labeling water molecules. Water has a small molecular size (∼18 da) and can cross the BBB paracellularly or through transcellular mechanisms of active cotransport and free diffusion across the endothelial membrane and facilitates diffusion through aquaporin‐4 (AQP4) water channels expressed in astrocytic endfeet.^17^, ^18^

**RESEARCH IN CONTEXT**

1. **Systematic review**: We reviewed the literature using online databases, including PubMed and Google Scholar, meeting abstracts, and preprint servers with a focus on in vivo imaging, CSF, and blood‐based biomarkers of BBB dysfunction. We focused primarily on studies conducted in humans, within the setting of aging and ADRD.
2. **Interpretation**: Since BBB dysfunction is an early mechanism in the development of ADRD, in vivo biomarkers are increasingly sought to measure BBB dysfunction in humans. Our review describes the current state of human BBB biomarkers, including their strengths and limitations and their implications in normal aging and ADRD.
3. **Future directions**: We discuss candidate blood‐based biomarkers of BBB dysfunction that warrant further investigation. Further studies incorporating a multimodal approach to the investigation of BBB integrity will provide meaningful information on the clinical utility of these biomarkers and provide tools to better understand vascular involvement in ADRD.

#### 2.1.1 Dynamic contrast‐enhanced MRI

Dynamic contrast‐enhanced (DCE) MRI collects dynamic images to measure the signal change following an intravenous injection of GBCA. Using pharmacokinetic modeling, the rate of GBCA extravasation from the bloodstream into the cerebral tissue is quantified as the transfer constant K_trans_.^19^ As GBCAs do not cross an intact BBB due to their large molecular size, an elevated K_trans_ is representative of a dysfunctional BBB. While this technique has been widely used to measure large changes in BBB permeability, such as in patients with tumors or strokes, an emphasis has been placed on developing DCE‐MRI techniques to measure subtle changes in BBB integrity, as seen in patients with cerebral small vessel disease (cSVD)^20^, ^21^, ^22^ and ADRD.^19^ Modifications to the acquisition and post‐processing methods can significantly improve the contrast‐to‐noise ratio for detection of small changes in K_trans_ and improve the accuracy of measurements.^23^, ^24^

BBB dysfunction measured by DCE‐MRI has been shown with normal aging,^7^, ^11^, ^25^ vascular disease,^8^, ^26^ mild cognitive impairment (MCI),^11^, ^27^ cSVD,^20^, ^21^, ^28^, ^29^, ^30^, ^31^ and AD.^32^, ^33^ Structural equation modeling shows that vascular risk factors such as hypertension increase whole‐brain K_trans_, which in turn is associated with white matter injury and decreased scores on tests of processing speed and executive function^34^; these data highlight a potential early mechanism behind the high rates of cognitive decline observed in the vascular population.^35^, ^36^ Regional analysis demonstrates that hippocampal K_trans_ is increased in individuals with MCI and AD, especially in carriers of the apolipoprotein E (*APOE)* ε4 allele.^11^, ^12^, ^33^, ^37^ Elevated hippocampal K_trans_ was independent of inflammatory status, vascular risk factors, or pathological markers of AD, which could signify earlier BBB disruption of relevance in neurodegeneration.^8^ Another study employing regional analysis of K_trans_ in patients with cSVD found that iron deposition in the temporal pole and caudate nucleus positively correlates with K_trans_ and cognitive decline.^31^ Altogether, these data suggest that BBB injury is a common mechanism in aging and ADRD that could promote cerebral accumulation of neurotoxic substances (i.e., iron) that may contribute to cognitive decline.^38^, ^39^, [Fig alz70104-fig-0001], [Fig alz70104-fig-0002]

In summary, DCE MRI is a widely available imaging technique with documented sensitivity to both large and subtle changes in BBB function in the setting of ADRD. However, extended scan times and larger sample sizes are needed to detect subtle BBB disruption with high accuracy, which is limited by patient tolerance, cost, and availability of scan time.^22^ There is also a need for a standardized protocol for optimal acquisition and data processing, as evidenced by high inter‐site variation in K_trans_, before DCE‐MRI can be utilized in a widespread clinical setting.^40^

#### 2.1.2 Dynamic susceptibility contrast

Dynamic susceptibility contrast (DSC) MRI is a technique used clinically to assess perfusion by capturing images at different time points during injection of GBCAs.^41^ DSC sequences can also be adapted to represent the leakage of GBCAs across a dysfunctional BBB. In patients with a dysfunctional BBB, GBCAs accumulate in the cerebral parenchyma, reducing the DSC MRI signal due to a T2* shortening.^42^ K2, a measure of BBB permeability, can be estimated using tissue with an intact BBB as a reference.^42^, ^43^, ^44^, ^45^, ^46^ K2 is positively associated with K_trans_,^47^, ^48^ suggesting that DSC and DCE techniques may be representative of similar measures of BBB dysfunction. However, quantification of K2 using DSC MRI has mainly been performed in subjects with a large degree of BBB disruption, such as those with a history of stroke or cancerous tumors.^47^, ^48^, ^49^, ^50^ These patients display elevated K2 values, representing increased distortion of the DSC signal due to elevated GBCA flow across the BBB.^42^, ^47^, ^49^, ^50^ While DSC MRI has potential to be a beneficial technique for measuring BBB dysfunction in human cognitive decline and dementia due to having significantly shorter scan times than DCE MRI, it is currently limited by low signal‐to‐noise ratios, and its ability to measure subtle BBB disruption has been questioned.^51^ Thus, DCE MRI remains the current standard for BBB evaluation with MRI. Future work and validation are warranted to improve the sensitivity of DSC MRI permeability metrics before they can be reliably utilized in the context of ADRD.

#### 2.1.3 Arterial spin labeling

Arterial spin labeling (ASL) is a non‐contrast‐based MRI technique used to quantify perfusion by magnetically labeling water molecules in flowing arteries. ASL can be used to measure BBB permeability (BBB ASL)^52^, ^53^ by modeling an intravascular (capillary) and extravascular (tissue) compartment. The most widely used techniques are diffusion‐prepared ASL (DP ASL)^54^ and multi‐echo ASL (ME ASL),^55^, ^56^ and both exploit the differing physical properties of blood and tissue to separate the compartments. The diffusion coefficient of water molecules in the blood is ∼100 times greater than that of the tissue and DP ASL uses a diffusion gradient preparation to derive the ratio of the intravascular and extravascular compartments and determine the water exchange rate (k_w_) between compartments.^52^ Alternatively, ME ASL uses the difference in transverse relaxation (*T*_2_) between the blood and tissue to calculate the water exchange time (T_ex_), which is the inverse of K_w_.^55^

Using DP ASL, K_w_ values have been found to be negatively associated with age^57^, ^58^ and positively associated with cerebrospinal (CSF) Aβ42 concentrations in cognitively unimpaired participants, indicating that early Aβ accumulation in the brain may be associated with BBB ASL measurements.^59^ This was confirmed by Uchida et al., who observed decreased K_w_ in *APOE* ε4 carriers and a negative correlation of K_w_ with iron concentrations and Aβ deposition (Pittsburgh Compound B [PiB] PET) in the frontal and medial temporal lobes, further supporting the hypothesis that iron and Aβ accumulation are related to a dysfunctional BBB.^38^, ^39^, ^60^, ^61^ In contrast to DP ASL, an increase in K_w_ was found in older adults with ME ASL (also reported as a decrease in T_ex_).^18^, ^62^, ^63^

In a recent study comparing different BBB ASL modalities in the same cohort, DP ASL and ME ASL methods yielded significantly different K_w_ values and had opposite correlations with age.^64^ DP ASL showed a non‐significant negative correlation, while ME ASL had a significant positive correlation with age.^64^ While acquisition parameters and modeling methods could contribute, the authors also proposed physiological changes in BBB permeability that might explain the discrepancy between ASL methods.^64^ Increased K_w_ could result from increased paracellular water transport via tight junction permeability, while decreased K_w_ could result from decreased transcellular water transport from the loss of AQP4 water channels.^65^ However, future studies utilizing CSF AQP4 concentrations are warranted to disentangle mechanisms governing the BBB ASL signal.

BBB ASL and DCE MRI may also measure different aspects of BBB breakdown, as they measure the permeability to different molecules. Contrast‐based approaches measure paracellular transport of gadolinium across the endothelium, representing the permeability of larger solutes. In contrast, ASL methods use MRI‐labeled water as an endogenous contrast, representing water permeability through the endothelium via numerous paracellular and transcellular transport mechanisms. Recent studies show that DP ASL K_w_ and DCE's K_trans_ are weakly correlated in a few distinct regions.^66^, ^67^ Ying et al. compared K_w_ and K_trans_ in subjects with different subtypes of cSVD, showing that individual cSVD subtypes were characterized by differential manifestations of BBB dysfunction.^66^ These data highlight the need for a multifaceted evaluation of BBB dysfunction.

Altogether, these data indicate that BBB ASL is a promising technique and could have relevance in aging and neurodegeneration. Despite this, BBB ASL is currently limited by extensive scan times and low signal‐to‐noise ratios. Like contrast‐enhanced methods of quantifying BBB dysfunction, there is a need for standardization of acquisition and post‐processing methods. As of yet, there is no consensus in the field regarding the best BBB ASL methodology. Coordinated efforts, such as the DEBBIE‐AD consortium,^68^ are under way to address these issues with large, multicenter prospective studies in aging and ADRD.

### 2.2 Positron emission tomography

Positron emission tomography (PET) imaging detects the decay of intravenously administered radiopharmaceuticals to deliver functional information about metabolic and physiological processes occurring in the brain. Specific radiopharmaceuticals can be used to target molecular transporters responsible for their transport across the BBB. Measures of uptake and permeability can then be calculated during post‐processing, enabling estimation of BBB dysfunction.^69^, ^70^, ^71^

#### 2.2.1 (R)‐[^11^C]verapamil

P‐glycoprotein (P‐gp) is an ATP‐binding cassette (ABC) efflux transporter protein expressed on the luminal side of endothelial cells.^72^ P‐gp plays a key role in maintaining homeostasis in the CNS through the transport of a broad spectrum of substances from the brain to the bloodstream.^73^, ^74^ Preclinical and *post mortem* studies show that Aβ is one of the substrates for P‐gp, suggesting that dysfunctional P‐gp‐mediated transport across the BBB may contribute to cerebral amyloid accumulation in AD.^75^, ^76^, ^77^, ^78^, ^79^ P‐gp function in vivo can be measured with low test–retest variability using (R)‐[^11^C]verapamil and PET, which passively diffuses across the BBB and acts as a substrate for P‐gp.^80^, ^81^ The retention of (R)‐[^11^C]verapamil in the brain is directly associated with dysfunctional P‐gp transport across the BBB. P‐gp function is impaired with age, as demonstrated by significantly increased volume of distribution of (R)‐[^11^C]verapamil in healthy older adults compared to younger adults.^80^ Another study demonstrated that P‐gp function was further impaired in AD patients compared to age‐matched controls without cognitive impairment, though no relationship was observed between (R)‐[^11^C]verapamil and [^11^C]PiB, a tracer for Aβ plaques.^81^ The authors attribute this lack of an association to a ceiling effect of [^11^C]PiB. Future studies should further explore the association between P‐gp function and amyloid accumulation in vivo by utilizing plasma or CSF Aβ42/40, which may be more sensitive to early changes in ADRD.^82^, ^83^

In summary, PET imaging allows for visualization and quantification of transport mechanisms relevant in BBB function. However, the potential for widespread clinical utilization of PET for BBB dysfunction is limited because of its use of ionizing radiation and cost. Another fundamental limitation of PET is a lack of spatial resolution relative to other imaging modalities. To combat this limitation, PET is often paired with computed tomography (CT) or MRI to obtain high‐resolution structural images and improve spatial resolution. This practice further raises the cost and/or radiation exposure of PET, decreasing the potential utility of this technique in evaluating the BBB in a clinical setting.

### 2.3 Cerebrospinal fluid biomarkers

CSF biomarkers are cost‐effective alternatives to imaging techniques in assessing BBB integrity. When the BBB is disrupted, specific proteins or molecules originating either from the bloodstream or cells of the NVU can appear in the CSF, providing an opportunity to obtain biochemical rather than spatial information related to BBB dysfunction. The most significant limitation to CSF biomarkers overall is that lumbar puncture is required, which is invasive and can be more difficult to perform in an aging population.

#### 2.3.1 CSF/Serum albumin ratio (Q‐Alb)

The most widely used fluid biomarker of BBB dysfunction is the CSF/serum albumin ratio (Q‐Alb). Since albumin originates from the blood and is not normally present at high concentrations in the CSF, Q‐Alb provides direct information regarding BBB permeability. Q‐Alb is positively correlated with age^84^ and is elevated in MCI,^11^ patients with vascular risk factors^85^, and several other neurodegenerative diseases such as Lewy body dementia (LBD), AD, Parkinson's disease, and vascular dementia (VAD),^10^, ^86^, ^87^, ^88^, ^89^ highlighting BBB dysfunction as a common mechanism in human aging, cognitive decline, and dementia. Despite these associations between Q‐Alb and neurodegenerative diseases, a recent study found no direct association between Q‐Alb and amyloid‐PET or *APOE* genotype, suggesting that this increase in Q‐Alb may occur independently of AD pathology or be reflective of BBB physiology less related to certain types/stages of neurodegeneration.^10^ One potential indirect mechanism of *APOE* ε4 on Q‐Alb was outlined in Bonomi et al., who showed that vascular risk factors increased Q‐Alb, leading to cognitive decline,^85^ specifically in *APOE* ε4 carriers.^90^ Furthermore, recent work has found no correlation between Q‐Alb and K_trans_, suggesting that they may measure different aspects of BBB injury.^33^, ^91^ While K_trans_ is associated with white matter injury, Q‐Alb is associated with biomarkers of inflammation, vascular remodeling, and endothelial activation.^10^, ^84^, ^91^, ^92^ These data demonstrate the heterogeneity of BBB pathophysiology and the physiological link between BBB dysfunction, endothelial activation, and neuroinflammation that promotes cognitive decline.^10^, ^91^, ^93^

#### 2.3.2 CSF/Serum immunoglobulin G ratio

CSF/serum ratios of immunoglobulin G (Q‐IgG) are used in a similar fashion to Q‐Alb, where a higher value can indicate increased BBB permeability.^94^, ^95^, ^96^, ^97^, ^98^, ^99^ Elevated Q‐IgG has been reported in VAD compared to patients with AD and controls.^94^, ^95^ Some have researchers have reported increased Q‐IgG in AD,^97^ while others have found no significant change^94^, ^95^, ^96^; this inconsistency could be due to the small sample sizes utilized in these studies. Still more, higher Q‐IgG is associated with atrophy of the middle temporal lobe and strongly correlates with Q‐Alb in AD patients.^98^, ^99^ Despite these data, Q‐IgG interpretation can be clouded by intrathecal antibody synthesis. Future studies with larger sample sizes are warranted to fully understand the relationship between Q‐IgG and other biomarkers of BBB integrity as well as its relevance in aging and ADRD.

#### 2.3.3 Platelet‐derived growth factor receptor‐β

Soluble platelet‐derived growth factor receptor‐β (PDGFRβ) is released during pericyte injury, a cell type of the NVU with a crucial role in maintaining normal BBB function through mechanisms including regulation of cerebral blood flow.^100^ Pericyte coverage is reduced by approximately 25% to 45% in the hippocampi and frontal lobes of patients with VAD and AD, and reduced pericyte coverage correlates with decreased tissue‐level expression of PDGFRβ.^101^, ^102^ PDGFRβ is shed from pericytes into the surrounding tissues in response to hypoxia and Aβ,^103^ which can be detected in both serum and CSF, helping to explain the inverse relationship between soluble PDGFRβ levels and pericyte coverage.^11^, ^12^, ^104^, ^105^, ^106^, ^107^, ^108^, ^109^ CSF PDGFRβ increases with age and is associated with tau pathology^33^, ^105^, ^107^, ^108^, ^109^ and neuroinflammatory^33^, ^104^, ^107^, ^110^ markers measured in CSF, independent of *APOE* status or Aβ42, and strengthens the effect of Aβ42 on tau hyperphosphorylation.^108^ This suggests that CSF PDGFRβ may represent BBB dysfunction related to neuroinflammation and pericyte loss. Recent evidence shows that elevated baseline CSF PDGFRβ is associated with increased hippocampal K_trans_ and predicts future cognitive decline in *APOE* ε4 carriers.^12^ Another study found a significant positive correlation between CSF PDGFRβ and Q‐Alb, but not between PDGFRβ and whole‐brain K_trans_, which may be due to an underpowered sample for these comparisons.^33^ Indeed, a recent study containing a larger sample size showed that CSF PDGFRβ increased linearly with the severity of BBB damage defined by Q‐Alb.^111^ Elevated CSF PDGFRβ is associated with decreased functional connectivity in the default mode network, a collection of brain regions that are both structurally and functionally affected by AD.^112^, ^113^, ^114^, ^115^ While pericyte loss leading to elevated PDGFRβ may initially be an age‐related process,^108^ the resulting damage to the BBB is associated with an increased risk of cognitive decline, especially in patients at the highest risk of ADRD.^12^

#### 2.3.4 Angiopoietin‐2

Angiopoietin‐2 (ANGPT‐2) is another biomarker thought to represent BBB injury.^116^ ANGPT‐2 is a regulator of endothelial permeability and angiogenesis, counteracting the activity of ANGPT‐1 and Tie2.^117^ ANGPT‐2 is released from endothelial cells in response to unfavorable conditions such as hypoxia,^118^ resulting in reduced structural integrity, pericyte loss, and increased permeability of the BBB.^116^, ^117^, ^119^, ^120^, ^121^ CSF ANGPT‐2 was found to be elevated in patients with MCI and AD, and had positive associations with tau pathology and CSF PDGFRβ.^116^ Furthermore, CSF ANGPT‐2 correlated positively with markers of neuronal injury (neurogranin and α‐synuclein), astrocytic neuroinflammation (YKL‐40), and BBB dysfunction (Q‐Alb).^116^ These data suggest that CSF ANGPT‐2 may be a biomarker of BBB dysfunction associated with neuroinflammation, endothelial activation, and pericyte loss, though further studies are needed to confirm this. Furthermore, the relationships between CSF ANGPT‐2 and longitudinal cognitive decline and *APOE ε4* carriership are still undetermined and warrant further exploration.

#### 2.3.5 YKL‐40

YKL‐40 is a glycoprotein secreted by many cell types, including macrophages, chondrocytes, endothelial cells, vascular smooth muscle cells, and various cancer cells.^122^, ^123^, ^124^, ^125^ YKL‐40 is abundantly expressed in reactive astrocytes during neuroinflammation.^126^, ^127^ CSF YKL‐40 increases with age, which occurs at a significantly higher rate in *APOE* ε4 carriers compared with non‐carriers.^128^ Interestingly, higher CSF YKL‐40 levels are associated with gray matter atrophy in APOE ε4 carriers, but not in non‐carriers. This suggests that while increases in CSF YKL‐40 are common as one ages, this process may be further exacerbated and detrimental in the setting of AD pathology.^128^, ^129^ This is further supported by data showing elevated CSF YKL‐40 levels in individuals with MCI and AD and significant relationships with CSF measures of tau pathology.^130^, ^131^, ^132^, ^133^, ^134^, ^135^ Furthermore, CSF YKL‐40 has been shown to positively correlate with pericyte loss, as measured by PDGFRβ.^104^ Altogether, these data indicate that elevated CSF YKL‐40 may serve as a biomarker of BBB dysfunction given the role of astrocytes in maintaining BBB integrity. Additional studies with larger sample sizes are warranted to further elucidate the relationship between CSF YKL‐40 and BBB dysfunction and its relevance in ADRD.

#### 2.3.6 Vascular‐endothelial cadherin

Vascular‐endothelial cadherin (VE‐Cadherin) is a calcium‐dependent cell adhesion receptor expressed exclusively on endothelial cells and has a critical role in regulating endothelial permeability and cell–cell communication through adherens junctions,^136^ angiogenesis,^137^ and leukocyte adhesion/extravasation.^138^ Recent studies utilizing both animal models and human tissue show reduced cerebral expression of VE‐Cadherin in AD brains, which translates to increased levels in CSF.^139^ Higher levels of tau and APOE found in AD brains have been associated with reduced cerebral expression of VE‐Cadherin in *post mortem* studies.^140^ Indeed, a large cross‐sectional ADRC study shows that CSF VE‐Cadherin levels are elevated in cognitively unimpaired patients with preclinical AD and correlates positively with CSF ADRD biomarkers including Aβ42/40, ptau‐181, total‐tau, and neurofilament light chain (NfL).^139^ Furthermore, CSF VE‐Cadherin levels are associated with worse scores on individual cognitive tests of executive function (Digit–Symbol), language (Animal Fluency), and episodic memory (FCSRT‐Free) among preclinical AD patients, even after adjusting for the effects of potential confounders such as age, sex, education, APOE ε4, and white matter injury.^139^ CSF VE‐Cadherin also partially mediates the effect of amyloid and tau on cognitive performance, supporting the hypothesis that endothelial dysfunction is relevant in early stages of neurodegeneration.^139^ While these data support the potential utility of CSF VE‐Cadherin in early AD, it has not been compared to other imaging or fluid biomarkers of BBB dysfunction; thus, additional studies are warranted to understand how CSF VE‐Cadherin relates to established biomarkers of BBB dysfunction and to confirm its role in the progression of ADRD.

#### 2.3.7 Aquaporin‐4

Aquaporin‐4 (AQP4) is a water channel that plays a key role in the clearance of solute from the cerebral parenchyma by facilitating the exchange of CSF for interstitial fluid, commonly referred to as the glymphatic system.^141^ AQP4 is mainly expressed in astrocytic endfeet,^142^ and *post mortem* studies have demonstrated that altered AQP4 expression is associated with AD.^143^ CSF AQP4 increases with age^144^ and is elevated in MCI and AD.^111^, ^144^, ^145^, ^146^ In addition, CSF AQP4 was shown to positively correlate with the severity of BBB dysfunction (as defined by Q‐Alb)^111^ and CSF biomarkers of tau pathology.^144^, ^145^, ^146^ Altogether, these data indicate that CSF AQP4 may be a useful biomarker of BBB dysfunction in ADRD related to astrocyte function. Future studies should place increased emphasis on comparing CSF AQP4 levels to ASL‐derived K_w_ and T_ex_ to further elucidate the mechanisms governing the ASL signals.

### 2.4 Future directions: blood‐based biomarkers of BBB/NVU integrity

Blood‐based biomarkers are beneficial due to their accessibility, affordability, and lack of invasiveness compared to CSF, contrast‐enhanced MRI, and PET biomarkers but tend to have limited or unknown sensitivity and specificity because they have mostly not been compared to established methodologies of quantifying BBB dysfunction. Promising candidates for detecting BBB dysfunction in peripheral blood include soluble factors that are related to various structural, cellular, or physiological components of the BBB.^106^, ^116^, ^147^ The development and validation of blood‐based biomarkers of BBB integrity would have meaningful clinical and experimental implications as a non‐invasive means of monitoring disease progression and BBB pathology in the primary and secondary care of patients with ADRD,^11^ stroke,^106^ and even COVID‐19.^148^, ^149^

#### 2.4.1 Glial fibrillary acidic protein

Glial fibrillary acidic protein (GFAP) is a biomarker of reactive astrocytosis, which is an evolutionarily conserved defensive mechanism for astrocytes.^150^ Reactive astrocytosis is characterized by an abnormal increase in the number of astrocytes in response to neuronal injury, neuroinflammation, and neurodegeneration.^151^, ^152^, ^153^ *Post mortem* studies have confirmed serum GFAP correlates with astrocyte reactivity in AD brains, suggesting that plasma GFAP is representative of NVU dysfunction.^147^ GFAP may be representative of BBB dysfunction because astrocytes are one of the major functional and structural units of the BBB, and their endfeet project directly onto the capillaries of the brain, providing a direct route for GFAP release into the bloodstream. In cognitively unimpaired adults, higher blood GFAP levels are associated with an increased risk for dementia and faster rate of cognitive decline.^154^, ^155^ Blood GFAP levels increase with aging and are further elevated in females, MCI, AD, Parkinson's disease, and frontotemporal dementia (FTD) but are not affected by the *APOE* ε4 allele.^147^, ^155^, ^156^, ^157^, ^158^, ^159^, ^160^, ^161^ Indeed, plasma GFAP levels have been shown to accurately differentiate amyloid positivity defined by PET imaging and CSF biomarkers, independent of *APOE* ε4 status.^155^, ^156^ Interestingly, recent studies have demonstrated that plasma GFAP is a better predictor of AD pathology than CSF GFAP; one reason that this could be the case is that plasma GFAP is indicative of astrocytosis severe enough to cause BBB damage/leakage.^156^, ^162^ However, despite the growing relevance of blood‐based GFAP levels as a biomarker of ADRD pathology, whether it is truly reflective of BBB damage/integrity remains unknown because it has not been directly compared to K_trans_ or other measures of BBB integrity. Additionally, GFAP is associated with damage in both the enteric^163^ and peripheral nervous system,^164^ which warrants further evaluation of the sensitivity and specificity of plasma GFAP for NVU dysfunction.

#### 2.4.2 YKL‐40

Similar to GFAP, YKL‐40 is abundantly expressed by reactive astrocytes, a major cell type of the BBB/NVU.^126^, ^127^ Plasma YKL‐40 increases with age and is elevated in a multitude of neurodegenerative diseases, including AD, LBD, and VAD.^165^, ^166^, ^167^, ^168^ A meta‐analysis of subjects who were non‐demented at baseline demonstrated that plasma YKL‐40 is associated with an increased risk for incident dementia, poorer cognition, and brain atrophy independent of age, sex, vascular risk factors, and *APOE* ε4.^168^ In addition, CSF and plasma YKL‐40 are significantly correlated, suggesting that plasma YKL‐40 is representative of NVU disruption occurring in the CNS.^165^ ^,^ ^170^ ^,^ ^171^ This association could represent a normal clearance mechanism, but as mentioned in the GFAP section, astrocytes project directly onto capillaries of the brain providing a route for entry into the bloodstream. Despite increasing data supporting the use of plasma YKL‐40 in ADRD, it is important to note that YKL‐40 is also expressed by many other cell types throughout the body and its interpretation could be altered by systemic inflammation and other processes.^122^, ^123^, ^124^, ^125^ Nonetheless, plasma YKL‐40 remains associated with poorer cognition and brain atrophy even after adjusting for peripheral (hs‐CRP) and systemic (cardiovascular risk factors) sources of inflammation.^168^ Although these data indicate that plasma YKL‐40 is representative of CNS pathology, implementing plasma YKL‐40 as a stand‐alone biomarker of BBB dysfunction is unlikely because of its low specificity. Instead, plasma YKL‐40 may be more useful as part of a larger panel of biomarkers related to BBB pathophysiology to investigate different pathways of BBB dysfunction; further studies are warranted to confirm this.

#### 2.4.3 Platelet‐derived growth factor receptor‐β

Pericytes, a crucial cell type of the BBB, release PDGFRβ into the surrounding tissues and bloodstream in response to injury.^103^ As pericytes are ∼100× more abundant in the CNS compared to the rest of the body,^1^, ^4^ PDGFRβ levels in serum may be indicative of pathology in the CNS. The use of serum PDGFRβ as a blood‐based biomarker for BBB injury is supported by studies that found significant positive associations between serum and CSF PDGFRβ levels, increasing the likelihood that serum PDGFRβ has a degree of specificity for pericyte injury occurring in the CNS.^105^, ^109^ A *post mortem* analysis found elevated serum PDGFRβ levels in individuals with a history of cerebrovascular events,^106^ a population known to have extensive BBB injury and exacerbated ADRD risk.^106^, ^170^, ^171^, ^172^ There are not yet published studies comparing blood PDGFRβ levels with other markers of BBB injury or AD pathology.

#### 2.4.4 Angiopoietin‐2

ANGPT‐2 is released by endothelial cells resulting in pericyte detachment and increased BBB permeability by antagonizing ANGPT‐1 and Tie2 activity.^117^ An analysis of cognitively unimpaired subjects with paired serum and CSF found positive relationships between serum ANGPT‐2 and CSF ANGPT‐2, as well as Q‐Alb.^116^ These relationships did not reach statistical significance, which may be attributed to a small sample size^116^; however, in our opinion, the data support the merit of further studies evaluating serum ANGPT‐2 as a blood‐based biomarker of BBB injury.

#### 2.4.5 Other putative blood‐based biomarkers of BBB dysfunction

There are several other blood‐based biomarkers that are being explored such as cell adhesion molecules^173^, ^174^, ^175^ (VCAM‐1, ICAM‐1, PECAM‐1), matrix metalloproteases^176^, ^177^, ^178^, ^179^ (MMP‐2, MMP‐9, MMP‐7), cytokines^84^, ^180^, ^181^ (IL‐6, TNF‐α, migration inhibitory factor), and more. We elected not to highlight these because they appear less specific for BBB pathology, and they have largely not been studied in the context of human aging and ADRD. As these biomarkers undergo further development, they may provide important information for broader pathophysiological understanding and serve as a basis for future studies.

## 3 CONCLUSION

DCE MRI is the current gold standard for detection of BBB dysfunction. DSC MRI and BBB ASL are promising techniques due to shorter scan times or not requiring GBCA injection, respectively, but they are limited by low signal‐to‐noise ratios, and additional work is needed to improve their utility. PET can be utilized to interrogate specific molecular transporters of the BBB but is limited by cost, exposure to ionizing radiation, and spatial resolution. CSF biomarkers are a cost‐effective alternative to MRI and PET and correlate well with ADRD pathology, though they are limited by the need for lumbar puncture. Promising candidate blood‐based biomarkers of BBB dysfunction exist; however, related studies have largely contained small sample sizes, and no study to our knowledge has compared these biomarkers to the current gold standards such as DCE MRI.

Of great importance, we find it possible, if not likely, that “BBB dysfunction” is a combination of multiple pathologies. With that in mind, as this field advances, it seems imperative to understand that both imaging and fluid biomarkers may represent different subtypes or pathways of BBB damage. We think comprehensive, longitudinal studies with an evaluation of the BBB incorporating multiple modalities will greatly benefit diagnostic capabilities and provide tools to better understand the underlying pathophysiology of ADRD related to BBB integrity.

## CONFLICT OF INTEREST STATEMENT

The authors declare that they have no conflicts of interest. Author disclosures are available in the supporting information.

## Supporting information



Supporting Information
